# The prognostic value of L1CAM in association with p53 in high-grade endometrial cancer

**DOI:** 10.3332/ecancer.2025.2011

**Published:** 2025-10-09

**Authors:** Eduardo Paulino, Guilherme Gomes de Mesquita, Andreia Cristina de Melo

**Affiliations:** 1 Brazilian National Cancer Institute, Rio de Janeiro, RJ 20220410, Brazil; 2 Oncologia D'OR, Rio de Janeiro, RJ 20220410, Brazil; 3 Grupo Oncoclinicas, Rio de Janeiro, RJ 20220410, Brazil; a https://orcid.org/0000-0003-1080-1058; b https://orcid.org/0009-0003-8737-9665; c https://orcid.org/0000-0002-1201-4333

**Keywords:** L1CAM, p53 aberrant, endometrial cancer

## Abstract

Endometrial cancer (EC) treatment changed substantially with the introduction of molecular classification. There is a paucity of data regarding the added value of L1CAM in patients with p53 aberrant tumours. The present study aimed to analyse the prognostic value of L1CAM associated with p53 aberrant EC. Patients with EC treated between 2010 and 2016 were retrospectively evaluated. Patients included in this analysis must have reviewed high-grade histologies (endometrioid grade 3, serous, clear cell, carcinosarcoma, mixed and undiffrentiated). Samples were subjected to immunohistochemistry for L1CAM and p53. Recurrence-free survival (RFS) and overall survival (OS) were analysed by the Kaplan-Meier method and log-rank test. Cox proportional hazards regression was performed for multivariable analysis. From 2010 to 2016, 464 patients met the inclusion criteria. Patients with p53 wild type and L1CAM negative (p53wt/L1CAMneg) corresponded to 13.6% (59 patients) of the population, p53 wild type and L1CAM positive (p53wt/L1CAMpos) to 11.7 % (51 patients), aberrant p53 and L1CAM negative (p53ab/L1CAMneg) to 32.9% (143 patients) and aberrant p53 with L1CAM positive (p53ab/L1CAMpos) to 41.8% (182 patients). In univariate and multivariate analysis, compared to patients with p53wt/L1CAMneg, the presence of p53wt/L1CAMpos, p53ab/L1CAMneg and p53ab/L1CAMpos was statistically associated with a worse RFS (HR 2.02; HR 2.20 and HR 2.99, respectively) and OS (HR 2.39; RH 2.31 and RH 2.94, respectively). In the present analysis of a high histological risk population, stages I–IV, we observed that the presence of p53ab/L1CAMpos was associated with a worse RFS and OS when comparing p53wt/L1CAMneg patients. Patients with L1CAMpos had the same worse prognosis as p53ab tumours.

## Introduction

Endometrial cancer (EC) is the most common gynecologic cancer in developed countries and is the only one with a rising incidence and mortality rate. It is expected to affect 142.687 patients in Europe in 2040 [1]. In some developing countries, the incidence is also rising due to the ageing and weighting of the population. Recently, the adjuvant treatment has changed dramatically with the inclusion of molecular classification. Patients with p53 aberrant tumours have a high rate of recurrence as well as poorer survival compared to other molecular groups (named *POLE* ultramutated, microsatellite instability and non-specific molecular profile) [2]. When the molecular classification is available and performed, it is recommended that all patients with p53 aberrant and myometrial invasion receive a combination of chemotherapy plus radiotherapy [3].

L1CAM is a transmembrane glycoprotein implicated in tumour growth and metastasis [4]. It has already been shown to be prognostic in retrospective cohorts of EC, especially in low-grade early-stage tumours, with patients having worse outcomes than patients without L1CAM expression [5, 6]. In high-grade and advanced EC, its prognostic value has been debated [7, 8]. Positive L1CAM expression is more common in high-grade histologies (especially non-endometroid EC), p53 aberrant tumours and advanced stages [7]. This research aimed to evaluate the prognostic value of L1CAM in association with p53 in a cohort of high-grade EC patients.

## Methods

This study was approved by the institutional ethical committee (approval number 26543019.5.0000.5274) and followed the Good Clinical Practices.

Patients with EC treated at the Brazilian National Cancer Institute (NCI) between 2010 and 2016 were retrospectively evaluated. Patients included in this analysis had high-grade histologies (endometrioid grade 3, serous, clear cell, carcinosarcoma, mixed and undifferentiated) and the treatment was performed at the Brazilian NCI. Clinical and pathologic characteristics such as age, race, performance status (PS), FIGO 2018 stage at diagnosis, surgical and adjuvant treatment (systemic or radiation therapy) were retrieved from the medical records. When the lymph node was not assessed in the surgical staging, patients were classified into the FIGO 2018 staging based on the pathological status of the uterine specimen.

All immunohistochemistry (IHC) analyses were performed on paraffin-embedded specimens cut into 4-μm sections and incubated at 60°C for at least 2 hours. Sections were deparaffinised with xylene and rehydrated. For antigenic recovery, the slides were placed in a steamer containing Trilogy™ (Cell Marque, Rocklin, CA, USA) solution for 30 minutes. Slides were allowed to cool to room temperature and then subjected to the IHC method using a peroxidase polymer-based commercial kit, according to the manufacturer's instructions (Novolink Max Polymer Detection System, Leica Biosystems). Slides were incubated either with anti-L1CAM (Clone 14.10, Biolegend) or anti-p53 (clone DO-7, Novocastra, Leica Biosystems), antibodies overnight at 4°C, both diluted at 1:1000. A reference sample for both antibodies (human breast sample for p53 antibody and human kidney sample for L1CAM antibody) was included in all routines as a positive control. As a negative control, the positive sample was assayed without the primary antibody. Counterstaining was performed with Harris hematoxylin solution. The expression of L1CAM in tumour cells was evaluated based on the degree of positivity. L1CAM-positive tumours were defined as more than 10% of tumour cells showing membranous L1CAM staining. Aberrant p53 expression was defined by one of the following: strong diffuse staining of 80%–100% of tumour cell nuclei (overexpression), complete absence of staining of tumour cell nuclei in the presence of positive internal control staining (null expression) or with abnormal cytoplasmic staining. Staining 1%–80% of nuclei, with variable intensity of staining, was considered wild-type p53 expression. The slides review and IHC for L1CAM and p53 were analysed on an optical microscope by the same experienced pathologist.

Disease progression was defined as pelvic, abdominal or distant progression. Pelvic included vaginal and local recurrences (including pelvic lymph nodes and local spread to the rectum and bladder); recurrences outside the pelvis, consisting of peritoneal carcinomatosis or omental metastasis, were classified as abdominal recurrences; distant recurrences include lung, liver, bone and brain metastases, as well as non-pelvic or para-aortic lymph node involvement. Simultaneous pelvic and abdominal recurrence was classified as abdominal recurrence; simultaneous pelvic and distant recurrence was considered distant recurrence, and simultaneous abdominal and distant recurrence was considered distant recurrence. Recurrence-free survival (RFS) was defined as the time from diagnosis to the date of confirmation of recurrence by imaging or clinically (local or distant) or death by any cause, with censoring of patients alive without recurrence. Overall survival (OS) was defined as the time from diagnosis to death, regardless of cause, with censoring of patients alive on the date of the last follow-up.

Patient and tumour characteristics were compared with the *t*-test for continuous variables and the *χ*^2^ statistic or Fisher's exact test for categorical variables. Rates of distant recurrences, locoregional recurrences, RFS and OS were analysed by the Kaplan-Meier method and log-rank test. Cox proportional hazards regression was performed for multivariable analysis. All statistical tests were two-sided; *p* values <0.05 were considered statistically significant. All tests were performed using the Stata software version 18.

## Results

From 2010 to 2016, 2,146 patients diagnosed with EC were enrolled at INCA and 464 patients met the inclusion criteria. The mean age was 65.8 (standard deviation (SD) 11.5) years, 44% were caucasian, 90.1% had ECOG PS of 0 or 1, 31.3% underwent total abdominal hysterectomy with or without bilateral salpingoophorectomy, 31.2% had endometrioid grade 3 and 25.4% serous histology, 50.8% received adjuvant treatment with chemotherapy followed or not by radiotherapy and 27% radiotherapy and/or brachytherapy. Most patients were stage I (44.6%) or III (30%). In 29 patients, it was not possible to perform IHC on one or both markers (L1CAM or p53); 327 (74.3%) patients had an aberrant p53 pattern and 236 (53.5%) were L1CAM positive. Patients with p53 wild type and L1CAM negative (<10%, p53wt/L1CAMneg) corresponded to 13.6% (59 patients), p53 wild type and L1CAM positive (>10%, p53wt/L1CAMpos) to 11.7% (51 patients), aberrant p53 and L1CAM negative (<10%, p53ab/L1CAMneg) to 32.9% (143 patients) and aberrant p53 with L1CAM positive (>10%, p53ab/L1CAMpos) to 41.8% (182 patients). Table 1 describes the main characteristics of the population.

Compared to p53wt/L1CAMneg, the p53ab/L1CAMpos pattern was associated with older age (67.9 versus 63.9 years), non-endometrioid histology (80.8% versus 59.3%), stage III/IV (53.3% versus 30.5%), more relapses (55.2% versus 22%) and deaths (68.7% versus 27.1%). The presence of p53wt/L1CAMpos and p53ab/L1CAMneg was also associated with worse prognostic features compared to p53wt/L1CAMneg. The site of relapse was not statistically different among the different patterns of p53/L1CAM, with the majority of relapses occurring at extrapelvic sites. Table 2 describes the association of the p53/L1CAM patterns with patients' characteristics and outcomes.

**Figure 1.**
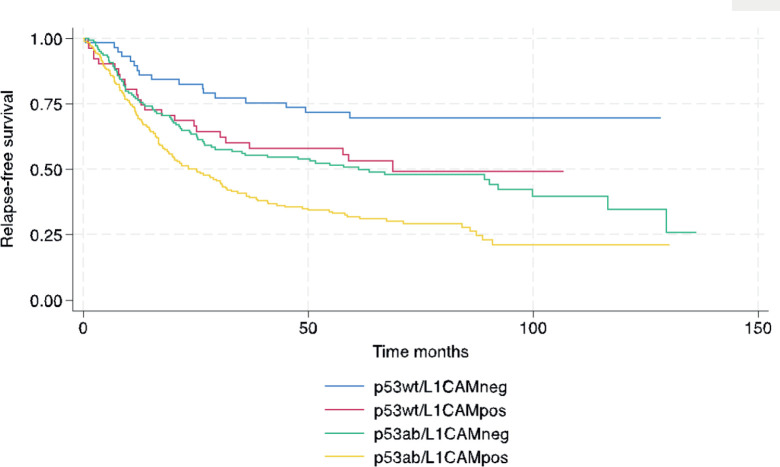
Relapse-free survival.

**Figure 2.**
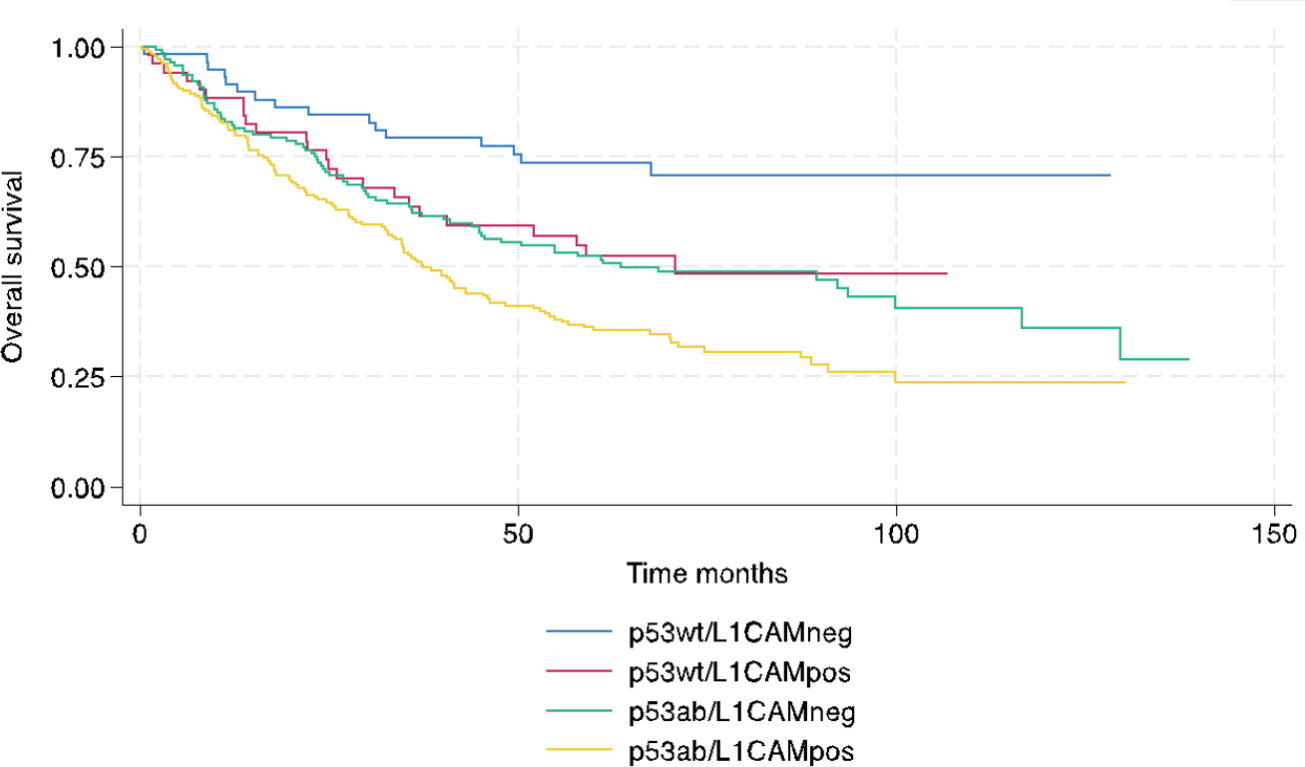
OS.

In terms of survival, the median follow-up for this cohort was 73.9 months. The median RFS and OS for the entire cohort were 38.4 and 53.5 months, respectively. The following variables were statistically associated with RFS in multivariable Cox regression analysis: age, PS, carcinosarcoma, FIGO stage, p53/L1CAM pattern and adjuvant treatment. For OS, the same variables were associated except for adjuvant radiotherapy. The L1CAM positive (p53wt/L1CAMpos) or aberrant p53 (p53ab/L1CAMneg) yields a worse prognosis compared to p53wt/L1CAMneg and the combination of p53ab/L1CAMpos yields an even greater risk of relapse (HR 2.99; 95% CI 1.74 to 5.13, *p* < 0.001) and death (HR 2.94; 95% CI 1.69 to 5.09, *p* < 0.001) (Table 3 and Figures 1 and 2).

## Discussion

The studies regarding L1CAM were mainly performed in a population of low-risk histologies and early-stage EC. Zeimet *et al* [5] conducted a multicentre retrospective study in stage I EC and showed that patients with L1CAM-positive tumours (17.7% of the cases) were associated with increased recurrence and lower OS. In another cohort, Bosse *et al* [9] retrospectively studied L1CAM in the PORTEC 1 and 2 randomised trials and showed a higher distant relapse as well as worse OS for L1CAM-positive tumours. In the retrospective analysis conducted by the European Network for Individualised Treatment of Endometrial Cancer Centres, the expression of L1CAM was found in 10% of the 935 stage I endometrioid ECs and was a strong predictor of poor outcome [7]. In the same study, L1CAM was associated with advanced stage, nodal involvement, high tumour grade, non-endometrioid histology, lymphovascular space invasion and distant recurrences. In the last decade, much attention was given to the molecular classification of EC and the poor prognostic value of p53 aberrant tumours. Since the value of L1CAM is not well studied in the presence of p53 aberrant tumours, we aimed to analyse the combination of L1CAM and p53 in a cohort of high-risk histologies.

L1CAM-positive tumours are overexpressed in patients with p53 aberrant tumours; however, it does not seem that L1CAM is a surrogate of p53 aberrant tumours. In the Van Gool *et al* [8] and Kommoss *et al* [10] studies, 64% and 81% of patients with p53 aberrant tumours expressed L1CAM positivity, respectively, while in patients with L1CAM positive 47% and 53.4% were p53 wild type. In the present analysis, it was 56% and 21%, respectively [8, 10]. However, 11% expressed only L1CAM positivity and 32% solely expressed p53 aberrant. In Van Gool's study, including high-risk patients (as handled in PORTEC-3) from the TRansPORTEC collaborating institutions, L1CAM-positive tumours (defined as >10%) did not predict prognosis, whereas an alternative threshold (>50%) did [8]. Interestingly, in the present study, L1CAM-positive tumours showed worse outcomes than p53wt/L1CAMneg, similar to the presence of p53 aberrant tumours and the combination of p53ab/L1CAMpos seems to have an increased risk of recurrence and death compared to the presence of only L1CAMpos or p53ab. Although we cannot change our current practice, this finding is of clinical relevance since patients with p53wt/L1CAMpos could receive more aggressive adjuvant treatment like p53ab tumours, as well it could also be attractive to develop anti-L1CAM target therapies. This finding should be validated in others cohorts.

This study has some weaknesses inherent to retrospective studies, such as information and collection biases, small number of patients included, confounding biases and changes in the treatment pattern during the years dissected. Another limitation was the fact that only one pathologist reviewed the slides as interobserver agreement regarding p53 is low in the literature. Furthermore, it was not possible to perform a complete molecular classification as recommended (with *POLE* gene mutation and MSI testing). However, we observed important strengths such as the number of patients included and the review of slides and immunohistochemical analysis by a pathologist dedicated to clinical research.

## Conclusion

In conclusion, in this high-risk histology cohort, patients with L1CAM positive had a worse prognosis similar to p53-aberrant tumours, and the combination of p53ab/L1CAMpos showed an even higher risk of progression and death compared to p53wt/L1CAMneg. This finding should be confirmed in other cohorts.

## Conflicts of interest

Eduardo Paulino: Astrazeneca, GSK, MSD, Abbivie, Gilead, ADIUM, Daiichi Sankyo

Andreia Cristina De Melo: grants or contracts from Amgen, AstraZeneca, Bristol Myers Squibb, Clovis Oncology, GSK, MSD, Novartis, Pierre Fabre, Regeneron Pharmaceuticals, Inc., and Roche, with payments made to the institution; and payment or honoraria for lectures, presentations, speakers’ bureaus, manuscript writing or educational events from Adium, AstraZeneca, Bristol Myers Squibb, Daiichi Sankyo, GSK, MSD, Novartis and Roche.

Guilherme Gomers de Mesquita: no conflicts of interest.

## Funding

There was no funding for this research.

## Figures and Tables

**Table 1.** Characteristics of the study population.

	*N* (%)
Total	464
Age (mean – SD)	65.8 (11.5)
Race	
Caucasian	207 (44.6%)
Non-Caucasian	257 (55.4%)
PS	
0	116 (25.4%)
1	295 (64.7%)
2	33 (7.2%)
3	12 (2.6%)
Type of surgery	
TAH	14 (3.0%)
TAH + BSO	131 (28.3%)
TAH + BSO + LN	156 (33.7%)
TAH + BSO + LN + Omentectomy	162 (35.0%)
Histology	
ECG3	145 (31.2%)
Serous	118 (25.4%)
Clear cell	44 (9.5%)
Carcinosarcoma	63 (13.6%)
Mixed	91 (19.6%)
SOE	3 (0.6%)
FIGO stage	
I	207 (44.6%)
II	58 (12.5%)
III	139 (30.0%)
IV	60 (12.9%)
Myometrial invasion	
<50%	236 (51.0%)
>50%	227 (49.0%)
p53 status	
Wild type	113 (25.7%)
Aberrant	327 (74.3%)
L1CAM status	
Negative	205 (46.5%)
Positive	236 (53.5%)
p53/L1CAM	
p53wt/L1CAMneg	59 (13.6%)
p53wt/L1CAMpos	51 (11.7%)
p53ab/L1CAMneg	143 (32.9%)
p53ab/L1CAMpos	182 (41.8%)
Treatment	
Observation	103 (22.2%)
CT with/without RT	235 (50.8%)
RT and/or BT	125 (27.0%)
Relapse	
No	259 (55.9%)
Yes	204 (44.1%)
Death	
No	198 (42.7%)
Yes	266 (57.3%)

Abbreviations: SD, standard deviation; TAH, total abdominal hysterectomy; BSO, bilateral salpingoophorectomy; LN, lymphadenectomy; ECG3, endometrioid carcinoma grade 3; CT, adjuvant chemotherapy; RT, adjuvant radiotherapy; BT, adjuvant brachytherapy

**Table 2.** Characteristics of the study population by p53/L1CAM status.

	p53/L1CAM					
	p53wt/L1CAMneg	p53wt/L1CAMpos	p53ab/L1CAMneg	p53ab/L1CAMpos	Total	*p* value
*N* (%)	59 (13.6%)	51 (11.7%)	143 (32.9%)	182 (41.8%)	435 (100.0%)	
Age (SD)	63.9 (10.836)	64.0 (9.056)	64.7 (12.943)	67.9 (11.301)	65.8 (11.680)	0.016
Race						
Caucasian	27 (45.8%)	20 (39.2%)	61 (42.7%)	80 (44.0%)	188 (43.2%)	0.908
Non-Caucasian	32 (54.2%)	31 (60.8%)	82 (57.3%)	102 (56.0%)	247 (56.8%)	
PS						
0	15 (25.9%)	14 (27.5%)	41 (29.1%)	38 (21.5%)	108 (25.3%)	0.917
1	38 (65.5%)	32 (62.7%)	84 (59.6%)	121 (68.4%)	275 (64.4%)	
2	4 (6.9%)	3 (5.9%)	12 (8.5%)	14 (7.9%)	33 (7.7%)	
3	1 (1.7%)	2 (3.9%)	4 (2.8%)	4 (2.3%)	11 (2.6%)	
Type of surgery						
TAH	1 (1.7%)	1 (2.0%)	5 (3.5%)	5 (2.8%)	12 (2.8%)	0.622
TAH + BSO	15 (25.4%)	11 (21.6%)	40 (28.0%)	58 (32.0%)	124 (28.6%)	
TAH + BSO + LN	23 (39.0%)	23 (45.1%)	50 (35.0%)	51 (28.2%)	147 (33.9%)	
TAH + BSO + LN + Omentectomy	20 (33.9%)	16 (31.4%)	48 (33.6%)	67 (37.0%)	151 (34.8%)	
Histology						
ECG3	24 (40.7%)	14 (27.5%)	60 (42.0%)	35 (19.2%)	133 (30.6%)	<0.001
Serous	4 (6.8%)	6 (11.8%)	20 (14.0%)	77 (42.3%)	107 (24.6%)	
Clear cell	5 (8.5%)	11 (21.6%)	14 (9.8%)	14 (7.7%)	44 (10.1%)	
Carcinosarcoma	11 (18.6%)	3 (5.9%)	25 (17.5%)	21 (11.5%)	60 (13.8%)	
Mixed	13 (22.0%)	16 (31.4%)	24 (16.8%)	35 (19.2%)	88 (20.2%)	
SOE	2 (3.4%)	1 (2.0%)	0 (0.0%)	0 (0.0%)	3 (0.7%)	
FIGO stage						
I	35 (59.3%)	20 (39.2%)	73 (51.0%)	65 (35.7%)	193 (44.4%)	0.019
II	6 (10.2%)	10 (19.6%)	20 (14.0%)	20 (11.0%)	56 (12.9%)	
III	12 (20.3%)	14 (27.5%)	35 (24.5%)	70 (38.5%)	131 (30.1%)	
IV	6 (10.2%)	7 (13.7%)	15 (10.5%)	27 (14.8%)	55 (12.6%)	
Myometrial invasion						
<50%	31 (52.5%)	23 (45.1%)	77 (53.8%)	89 (48.9%)	220 (50.6%)	0.678
>50%	28 (47.5%)	28 (54.9%)	66 (46.2%)	93 (51.1%)	215 (49.4%)	
Treatment						
Observation	16 (27.1%)	7 (13.7%)	36 (25.4%)	37 (20.3%)	96 (22.1%)	0.055
CT with/without RT	24 (40.7%)	31 (60.8%)	61 (43.0%)	105 (57.7%)	221 (50.9%)	
RT and/or BT	19 (32.2%)	13 (25.5%)	45 (31.7%)	40 (22.0%)	117 (27.0%)	
Relapse						
No	46 (78.0%)	33 (64.7%)	88 (61.5%)	81 (44.8%)	248 (57.1%)	<0.001
Yes	13 (22.0%)	18 (35.3%)	55 (38.5%)	100 (55.2%)	186 (42.9%)	
Site of relapse						
Pelvic	4 (30.8%)	4 (22.2%)	21 (38.2%)	29 (29.0%)	58 (31.2%)	0.701
Abdominal	5 (38.5%)	5 (27.8%)	12 (21.8%)	24 (24.0%)	46 (24.7%)	
Distant	4 (30.8%)	9 (50.0%)	22 (40.0%)	47 (47%)	82 (44.1%)	
Death						
No	43 (72.9%)	27 (52.9%)	64 (44.8%)	57 (31.3%)	191 (43.9%)	<0.001
Yes	16 (27.1%)	24 (47.1%)	79 (55.2%)	125 (68.7%)	244 (56.1%)	

Abbreviations: SD, standard deviation; TAH, total abdominal hysterectomy; BSO, bilateral salpingoophorectomy; LN, lymphadenectomy; ECG3, endometrioid carcinoma grade 3; CT, adjuvant chemotherapy; RT, adjuvant radiotherapy; BT, adjuvant brachytherapy

**Table 3.** Multivariable analysis for survival (relapse-free and OS).

	Relapse free survival			OS		
Variables	HR	95% CI	*p*-value	HR	95% CI	*p*-value
Age	1.02	1.01–1.03	0.00	1.02	1.00–1.03	0.01
Race						
Caucasian	1	.	.	1	.	.
Non-Caucasian	1.11	0.85–1.46	0.45	1.15	0.87–1.51	0.33
PS						
0	1	.	.	1	.	.
1	2.16	1.47–3.16	0.00	2.50	1.66–3.75	0.00
2	2.50	1.45–4.32	0.00	3.45	1.97–6.03	0.00
3	3.27	1.50–7.13	0.00	4.12	1.84–9.21	0.00
Histology						
EG3	1	.	.	1	.	.
Serous	1.13	0.76–1.69	0.53	1.22	0.82–1.83	0.32
Clear cell	1.09	0.66–1.79	0.74	1.13	0.68–1.88	0.63
Carcinosarcoma	2.39	1.55–3.67	0.00	2.56	1.66–3.94	0.00
Mixed	0.94	0.61–1.43	0.76	0.89	0.57–1.37	0.59
SOE	0	.	.	0	0	1.00
Type of surgery						
TAH	1	.	.	1	.	.
TAH + BSO	0.84	0.37–1.90	0.67	0.72	0.31–1.65	0.44
TAH + BSO + LN	0.70	0.30–1.61	0.40	0.57	0.25–1.31	0.18
TAH + BSO + LN + Omentectomy	0.61	0.27–1.41	0.25	0.48	0.21–1.12	0.09
FIGO stage						
I	1	.	.	1	.	.
II	2.96	1.91–5.48	0.00	2.90	1.87–4.50	0.00
III	5.98	3.93–9.10	0.00	6.16	3.98–9.53	0.00
IV	12.89	7.9420.93	0.00	10.52	6.48–17.09	0.00
p53/L1CAM						
p53wt/L1CAMneg	1	.	.	1	.	.
p53wt/L1CAMpos	2.02	1.06–3.84	0.03	2.39	1.24–4.62	0.01
p53ab/L1CAMneg	2.20	1.28–3.77	0.00	2.31	1.33–4.02	0.00
p53ab/L1CAMpos	2.99	1.74–5.13	0.00	2.94	1.69–5.09	0.00
Treatment						
Observation	1	.	.	1	.	.
CT with/without RT	0.37	0.25–0.57	0.00	0.35	0.23–0.53	0.00
RT and/or BT	0.54	0.36–0.83	0.00	0.65	0.43–0.99	0.05

Abbreviations: SD, standard deviation; TAH, total abdominal hysterectomy; BSO, bilateral salpingoophorectomy; LN, lymphadenectomy; ECG3, endometrioid carcinoma grade 3; CT, adjuvant chemotherapy; RT, adjuvant radiotherapy; BT, adjuvant brachytherapy
